# Sequence-based Prediction of the Cellular Toxicity Associated with Amyloid Aggregation within Protein Condensates

**DOI:** 10.1021/acs.biochem.2c00499

**Published:** 2022-11-07

**Authors:** Attila Horvath, Michele Vendruscolo, Monika Fuxreiter

**Affiliations:** † John Curtin School of Medical Research, The Australian National University, Acton, ACT 2601, Canberra2600, Australia; ‡ Centre for Misfolding Diseases, Yusuf Hamied Department of Chemistry, University of Cambridge, CambridgeCB2 1EW, UK; § Department of Biomedical Sciences, University of Padova, Padova, PD35131Italy; ∥ Department of Physics and Astronomy, University of Padova, Padova, PD35131Italy

## Abstract

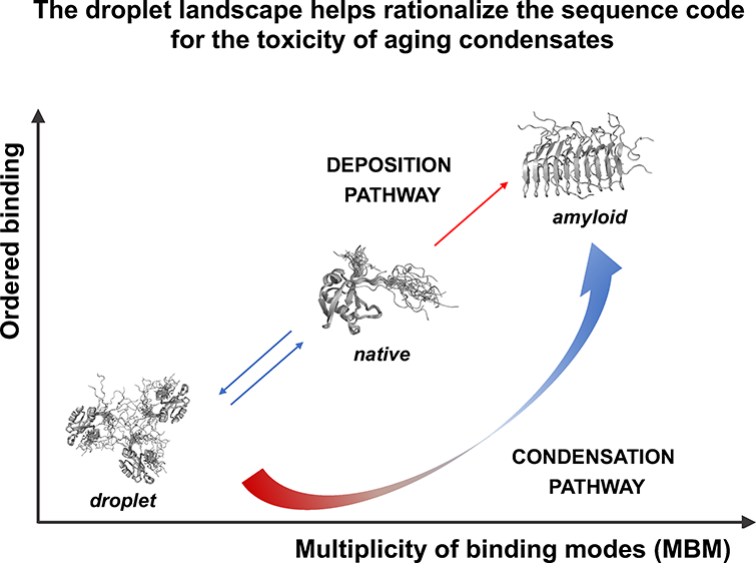

Various neurological dysfunctions are associated with cytotoxic amyloid-containing aggregates formed through the irreversible maturation of protein condensates generated by phase separation. Here, we investigate the amino acid code for this cytotoxicity using TDP-43 deep-sequencing data. Within the droplet landscape framework, we analyze the impact of mutations in the amyloid core, aggregation hot-spot, and droplet-promoting residues on TDP-43 cytotoxicity. Our analysis suggests that TDP-43 mutations associated with low cytotoxicity moderately decrease the probability of droplet formation while increasing the probability of multimodal binding. These mutations promote both ordered and disordered binding modes, thus facilitating the conversion between the droplet and amyloid states. Based on this understanding, we develop an extension of the FuzDrop method for the sequence-based prediction of the cytotoxicity of aging condensates and test it over 20,000 TDP-43 variants. Our analysis provides insight into the amino acid code that regulates the cytotoxicity associated with the maturation of liquid-like condensates into amyloid-containing aggregates, suggesting that, at least in the case of TDP-43, mutations that promote aggregation tend to decrease cytotoxicity, while those that promote droplet formation tend to increase cytotoxicity.

## Introduction

Increasing evidence demonstrates that proteins can populate three fundamental states in the cellular environment. In addition to the native and amyloid states,^1,2^ proteins can sample a dense, liquid-like state, which is reversibly formed from the native state through a phase separation process and is prone to irreversibly maturate into the amyloid state.^3−5^ This liquid-like state, also known as the droplet state, seems to be generally accessible to proteins^6^ and associated with a wide range of physiological processes that involve the formation of multicomponent functional assemblies referred to as membraneless organelles.^7,8^

**Figure 1:**
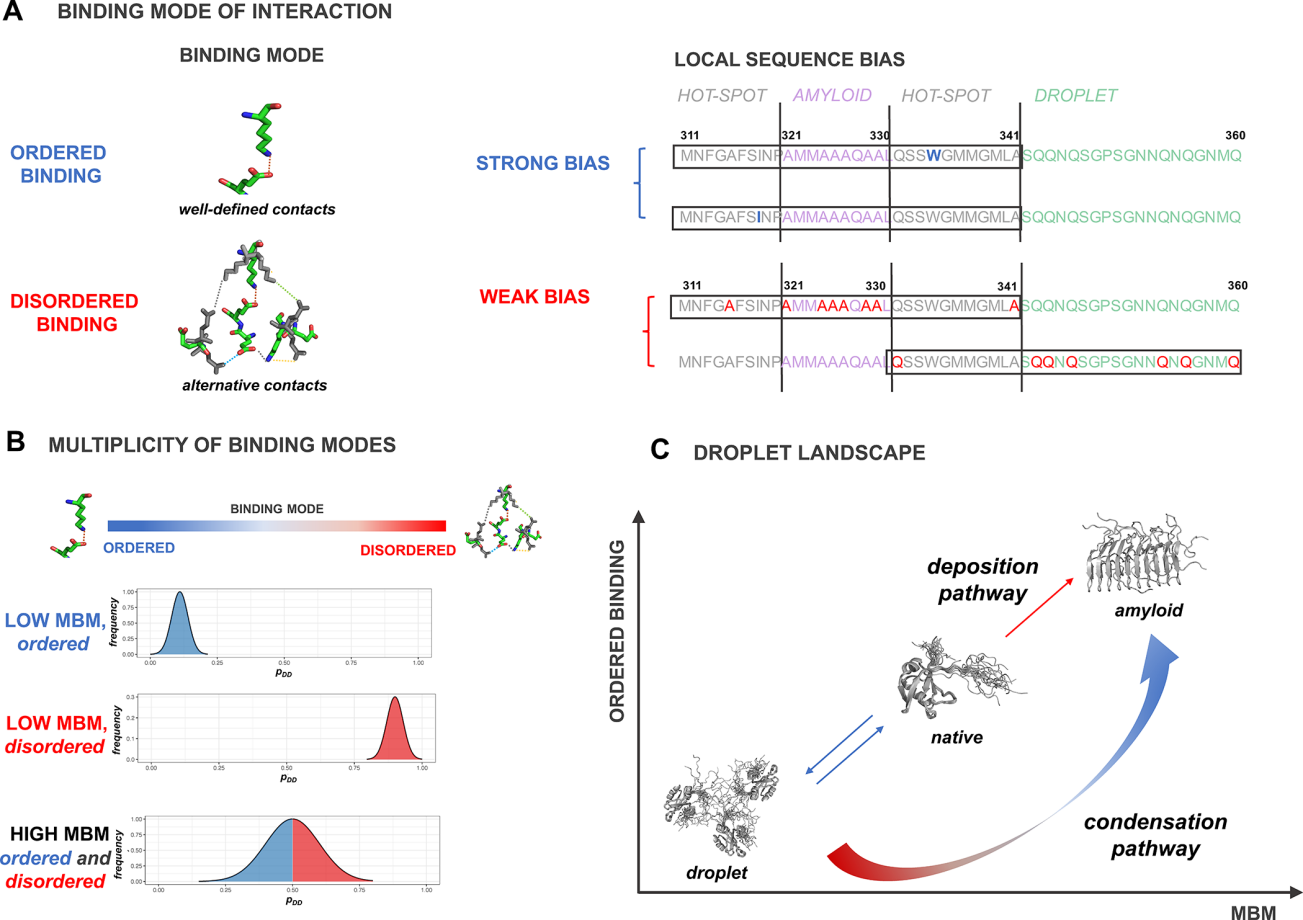
Interaction modes in the droplet and amyloid states of proteins. (A) The local sequence bias of a protein region determines its binding mode. Illustration of residues in regions with strong (blue) and weak (red) local sequence bias in the droplet (green), amyloid (purple), and hot-spot (gray) regions of TDP-43.^19^ The local sequence bias of a given region is defined based on the difference between the amino acid composition of the region itself and its flanking sequences.^20^ A strong bias leads to ordered binding modes with a well-defined contact pattern (orange dotted line). A weak bias leads to alternative contact patterns between similar sites of the same set of residues (colored dotted lines). (B) The multiplicity of binding modes (MBM) quantifies the spectrum of interaction modes sampled in the bound state. Protein interactions sample a wide range of binding modes from ordered (blue) to disordered (red). The MBM is low when only one of these modes is sampled. This means that in the bound state, the protein exhibits well-defined contact patterns (ordered binding, blue) or variable, heterogeneous binding patterns (disordered binding, red), but it is unlikely to switch between these scenarios. In the case of high MBM, a protein region can exhibit both types of interactions and can switch between ordered and disordered binding modes, depending on its partner, post-translational modification, or other cellular conditions. (C) Landscape of binding modes of the three main cellular states of proteins. The *x* axis reports the multiplicity of binding modes (MBM), and the *y* axis reports the ordered binding modes. The droplet state is characterized by disordered interactions (low degree of ordered binding) as well as low MBM. That is, residues promoting the droplet state unlikely change disordered to ordered interactions. In contrast, residues that form the amyloid state tend to exhibit ordered binding mode and high MBM, since they can change between disordered and ordered binding. The amyloid state can be formed through the deposition pathway (diagonal) through unfolding of the native state or through the condensation pathway (along the arrow passing through the lower-right corner) of irreversible maturation of the droplet state.

The droplet state is tightly regulated by the protein homeostasis system,^9^ including molecular chaperones,^10^ ubiquitin ligases, and the autophagy system.^11^ Upon dysregulation of the balance between the native state and the droplet state, however, the latter can evolve into the amyloid state (Figure 1).^12^ During this process of amyloid formation, known as the condensation pathway, cytotoxic intermediates can be generated.^13−17^ Cytotoxicity can be caused by a wide variety of molecular mechanisms, including protein mislocalization, a lack of availability of functional partners, or a presence of nonphysiological partners, and by changes in the protein structure, leading to oligomerization. In addition, a delayed reconversion to the native state of proteins trapped in a gel-like form can be due to recruitment of other cellular components.^18^

We previously investigated the amino acid code that determines the condensation pathway to amyloid formation.^21^ We suggested that the conversion of the droplet state to the amyloid state can be described by a droplet landscape based on the observation that the droplet and amyloid states are stabilized by different binding modes.^21,22^ The droplet state is mostly stabilized by disordered interactions, comprising heterogeneous binding patterns among the same residues (Figure 1A). The amyloid state, in contrast, is stabilized by ordered interactions, which are formed by well-defined contacts between residues (Figure 1A).

An important role in this discussion is played by protein regions that can sample both ordered and disordered binding modes. Regions that sample a multiplicity of binding modes (MBM), including both disordered and ordered interactions (Figure 1B), can be identified based on their sequences.^22^ Our analysis suggests that regions that change their binding modes upon alterations in the cellular environment overlap with regions becoming the amyloid cores of the condensates and thus can serve as aggregation hot-spots.^21^ Based on this insight, we could discriminate between FUS mutations associated with amyotrophic lateral sclerosis (ALS) and those not associated with the disease.^23,24^ In particular, our analysis indicates that disease-associated mutations increased the multiplicity of binding modes, i.e., the probability of sampling both ordered and disordered interactions, while similar mutations not affecting this property tended to be not associated with ALS.^21^

Here, we investigated the amino acid code that determines cytotoxicity of the aging condensates using deep-sequencing data of >20,000 mutants of the TAR binding protein 43 (TDP-43).^19^ The presence of neuronal aggregates of mutant TDP-43 is a molecular hallmark of ALS.^17,25,26^ The cytotoxicity of TDP-43 mutants measured in yeast indicated that mutations that enhanced amyloid formation decreased cytotoxicity,^19^ while mutations that affected the secondary structure increased cytotoxicity. By extending previous results on ALS-associated mutations,^23,24^ our analysis here indicates that TDP-43 mutants increase the probability of sampling both ordered and disordered interactions and in particular increase the multiplicity of binding modes of droplet-promoting residues. Based on this understanding, we report an extension of the FuzDrop method^6^ for the sequence-based prediction of the cytotoxicity of aging condensates.

## Results

### Aggregation Is Induced by Mutations Affecting the Local Sequence Bias

We previously reported the prediction of whether a protein region forms ordered or disordered interactions based on its local sequence bias^20^ (Figure 1A). The local sequence bias of a region arises from a difference in the amino acid composition between the region itself and its flanking sequences. When the bias is large, a well-defined interaction tends to be established through an ordered binding mode^20^ (Figure 1A,B). This type of interaction is usually established in specific complexes of proteins with sequences of high complexity. In contrast, when the bias is small, disordered interactions are promoted due to competing binding sites with similar properties^20^ (Figure 1A). In this case, a variety of alternative interaction patterns can be established among the same set of residues, resulting in a heterogeneous bound state (Figure 1A,B). Disordered binding modes are usually linked with low-complexity (LC) sequences and/or structural disorder.^27^ We previously reported that the droplet state is driven by such disordered interactions,^6^ which can be formed through a wide range of sequence motifs^5^ of aromatic, charged, and hydrophobic contacts.^28^

Thus, based on the local sequence bias, sequence elements promoting droplet formation and amyloid formation can be identified. Based on these insights, one can predict that aggregation could be initiated by sequence elements where mutations, post-translational modifications, or interactions with other cellular factors considerably increase the sequence bias and promote ordered interactions (Figure 1B,C).

### Droplet Landscape Representation of Aggregation within Protein Condensates

**Figure 2:**
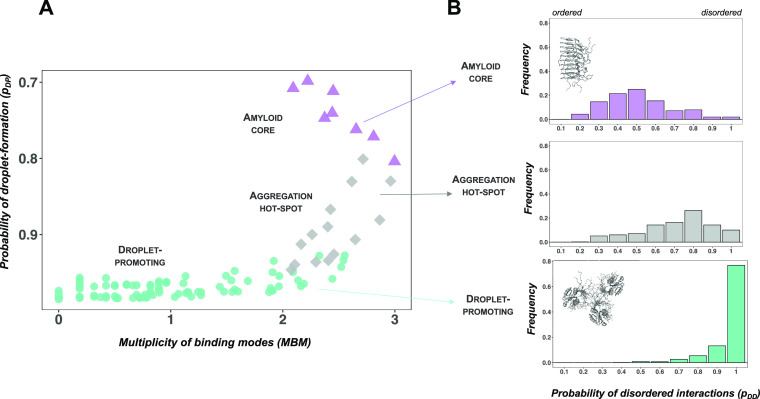
Droplet landscape representation of the LC region of TDP-43. (A) Droplet landscape of the LC region of TDP-43 (residues 262–414). The *x* axis of the droplet landscape corresponds to the multiplicity of binding modes (MBM).^22^ The *y* axis of the droplet landscape is defined by the droplet-promoting propensity (*p*_DP_).^6^ The droplet landscape of the wild-type TDP-43 LC domain (residues 262–414) illustrates droplet-promoting residues (residues 262–311 and 342–414, green circles) in the lower-left section of the droplet landscape with high *p*_DP_ values and low MBM values. In contrast, residues forming the amyloid core (residues 321–330, purple triangles) are in the upper-right section, exhibiting low *p*_DP_ values and high MBM values. Regions that readily convert into amyloids (i.e., “aggregation hot-spots”, residues 312–320 and 331–341, gray diamonds^19^) are in the lower cross section, with high *p*_DP_ values and high MBM values. These residues prefer disordered binding configurations, as well as also sample ordered states, as reflected by high interaction multimodality. (B) Frequencies of different binding modes in the TDP-43 LC domain. The frequencies of different binding modes from ordered to disordered interactions are shown for the amyloid core (purple), aggregation hot-spot (gray), and droplet-promoting region (green). While the amyloid core and the aggregation hot-spot exhibit wide distributions by sampling both the ordered and disordered interactions, leading to high MBM, the droplet region mostly samples disordered (unimodal) interactions, leading to low MBM.

As noted above (Figure 1C), to describe the transition from the droplet to the amyloid state, one can use a droplet landscape (Figure 2A). This landscape helps understand how the local sequence bias can be modulated by the cellular conditions, which cause a change in binding modes, thus leading to the conversion between the droplet and amyloid states.^21^ The *x* axis of the droplet landscape corresponds to the multiplicity of binding modes (MBM), which is computed based on the Shannon entropy of binding modes (*S*_bind_), as defined in the FuzPred method^22^ (Figure 2A). The *y* axis of the droplet landscape is defined by the residue-specific droplet-promoting propensity (*p*_DP_) of the FuzDrop method, which characterizes the likelihood for spontaneous phase separation^6^ (Figure 2A).

In the droplet landscape representation, droplet-promoting residues are found in the lower-left section of the droplet landscape (the “droplet region”), as they have a high probability to form the droplet state mostly by disordered interactions. Thus, the multiplicity of binding modes of the droplet regions is low (Figure 2A,B). By contrast, the amyloid-promoting residues are in the upper-right section of the droplet landscape (the “amyloid region”), as they have lower probability to undergo phase separation, by simultaneously sampling both ordered and disordered interactions. Thus, residues in the amyloid regions have a high multiplicity of binding modes^29^ (Figure 2A,B). Residues in the lower-right section of the droplet landscape have a high probability to form the droplet state and sample both ordered and disordered interactions. Therefore, these residues can initiate aggregation in the protein condensates (“aggregation hot-spot”) (Figure 2A). Hot-spot residues have a high multiplicity of binding modes, as they sample both ordered and disordered interactions (Figure 2B).

The methods to compute the values of the multiplicity of binding modes (MBM) and residue-specific droplet-promoting propensity (*p*_DP_) have been published previously^6,22^ and are publicly available.^30^ The *p*_DP_ values are computed using a binary logistic model (Methods, eq. 4) and a scoring function based on the conformational entropy of the free and bound states (Methods, eq. 5). This method was shown to be robust to identify droplet-promoting regions under physiological conditions.^6^

The droplet landscape of the wild-type TDP-43 LC domain (residues 262–414) illustrates distinct features of regions promoting droplet formation and amyloid formation and serves as hot-spots for aggregation (Figure 2A). Droplet-promoting residues (residues 262–311 and 342–414) are in the lower-left section of the droplet landscape and have high *p*_DP_ values and low MBM values. In contrast, residues forming the amyloid core (residues 321–330^31,32^) are in the upper-right section, exhibiting low *p*_DP_ values and high MBM values. Regions that readily convert to amyloids (i.e., “aggregation hot-spots”; residues 312–320 and 331–341^19^) are in the lower cross section with high *p*_DP_ values and high MBM values. These residues prefer disordered binding configurations, as well as also sample ordered states, as reflected by high MBM (Figure 2A,B).

### TDP-43 LC Mutations Increase the MBM of Droplet-Promoting Residues

**Figure 3:**
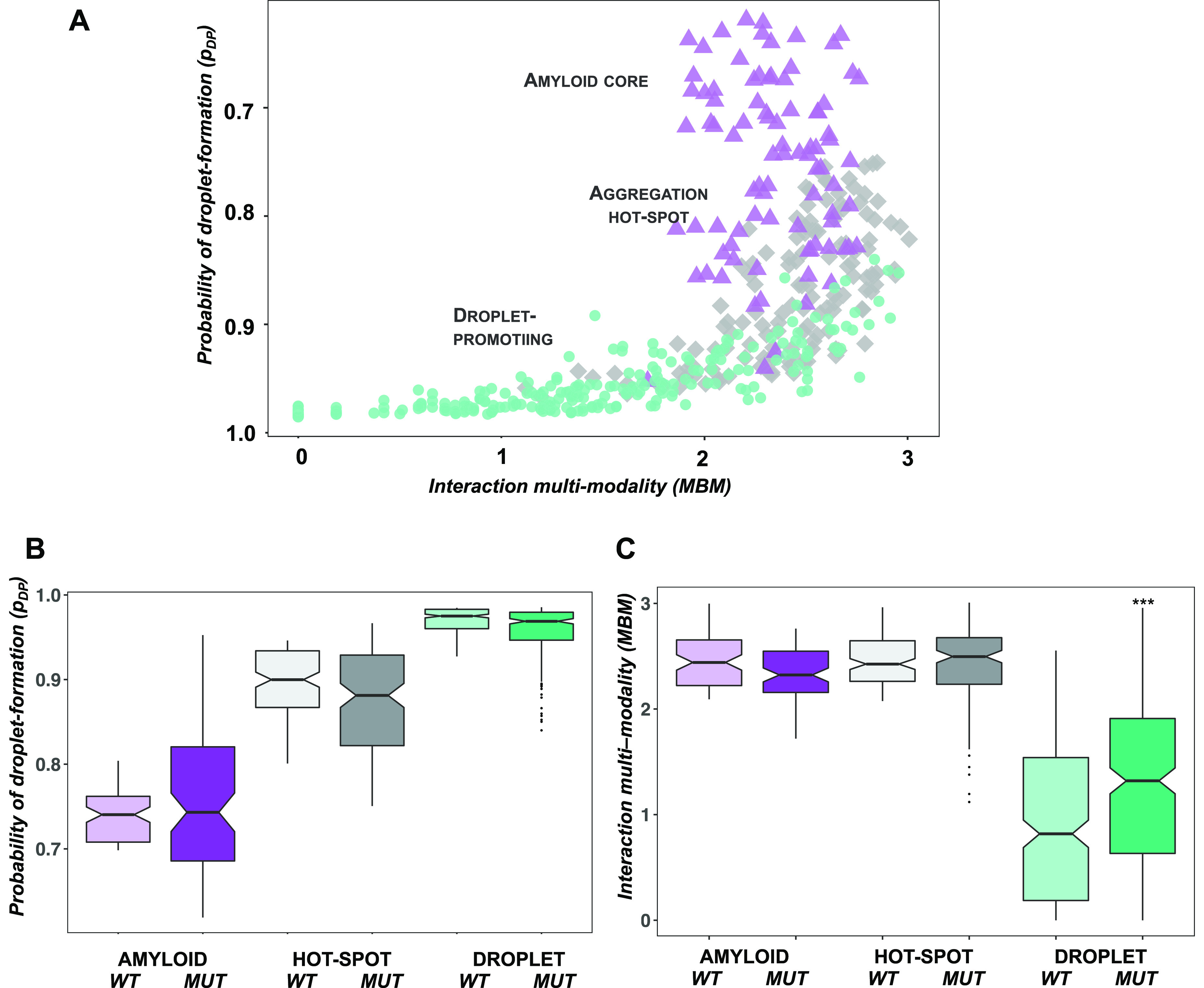
Single mutations increase the MBM of the TDP-43 LC domain. We analyzed 498 single mutations with a change in cytotoxicity (Δ*e*_tox_) > 3σ .^19^ (A) Droplet landscape of TDP-43 single mutants. Amyloid core residues (residues 321–330, purple triangles) and droplet-promoting residues (residues 262–311 and 342–414, green circles) considerably overlap with the aggregation hot-spot region (residues 312–320 and 331–341, gray diamonds). This indicates a high probability to phase separate (*p*_DP_ values, *y* axes) and a high multiplicity of binding modes (MBM, *x* axis) that reflects sampling both disordered and ordered interactions. (B) Comparison of droplet propensities of wild-type and mutant TDP-43 residues. No significant change was calculated between the phase separation probability of wild-type (light) and mutant residues (dark) in the amyloid core (purple), aggregation hot-spot (gray), and droplet region (green). (C) Comparison of MBM of wild-type and mutant TDP-43 residues. Mutations in the droplet region (dark green) significantly (*p* < 10^–3^) increase the MBM as compared to the wild-type values (light green), reflecting a shift in binding modes toward ordered interactions. The statistical significance was computed by the Mann–Whitney test of the R program.

A deep mutagenesis approach was recently reported to generate >50,000 TDP-43 variants.^19^ The cytotoxicity of these variants was assessed by monitoring growth rates in *Saccharomyces cerevisiae*, and mutants decreasing the growth rate were considered cytotoxic.^19^ We analyzed the impact of these mutations on the droplet landscape of the droplet region, amyloid core, and aggregation hot-spot residues of 498 single mutants, leading to a large change in cytotoxicity (Δ*e*_tox_ > 3σ, where σ is the average change over all the mutations;^19^Figure 3). Both the amyloid core and the droplet region are shifted toward the aggregation hot-spot region, with high *p*_DP_ values (≥0.75) and high MBM values (>2.25) (Figure 3A). That is, both the amyloid core and droplet-promoting residues could sample both ordered and disordered interactions, thus facilitating the conversion between these states. Along these lines, our analysis indicated that while the *p*_DP_ values did not significantly change for the droplet, amyloid, and aggregation hot-spot regions (Figure 3B), the MBM values significantly increased for the droplet region (Figure 3C).

### Cytotoxicity of TDP-43 Mutants Is Linked with Droplet Formation

**Figure 4:**
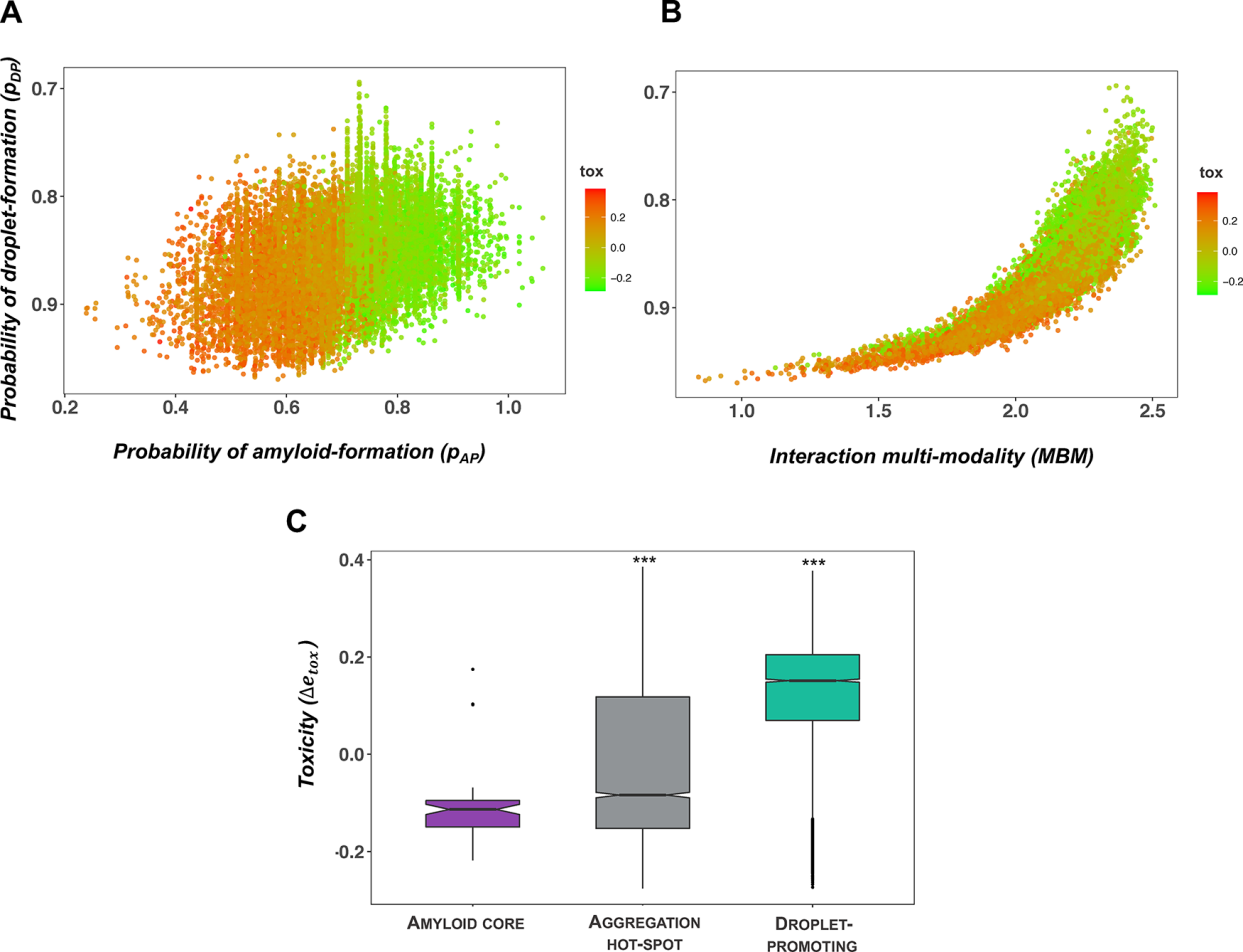
Investigation of the amino acid code of the cytotoxicity of TDP-43 LC double mutants. (A) Amyloid formation propensity is linked with a reduction of the cytotoxicity of TDP-43 LC double mutants. Variants with increased droplet-forming probabilities are more toxic than variants that tend to form amyloids. The cytotoxicity scale ranges from green (not cytotoxic) to red (cytotoxic) and is shown on the right. (B) Droplet formation propensity is linked with the cytotoxicity of TDP-43 LC double mutants. Droplet-promoting residues located at the left bottom of the droplet landscape (high *p*_DP_ and low MBM values) exhibit higher cytotoxicity than amyloid-promoting residues at the top right with lower *p*_DP_ and high MBM values. The cytotoxicity scale, which ranges from green (not toxic) to red (toxic), is shown on the right. (C) Cytotoxicity is linked with high droplet formation propensity. Variants were classified based on their position on the droplet landscape: droplet-promoting (*p*_DP_ > 0.85; MBM < 2.0; green), amyloid-promoting (*p*_DP_ < 0.75; MBM > 2.25; purple), and aggregation hot-spot (0.75 < *p*_DP_ < 0.85; MBM > 2.25; gray). The variants promoting droplet formation are significantly (*p* < 10^–6^) more toxic than amyloid-promoting variants and aggregation hot-spots. Cytotoxicity values were taken from ref (19).

Then, we analyzed how molecular determinants of the condensation pathway (i.e., the conversion from droplet to the amyloid state^21^) are related to cytotoxicity (Tables S1 and S2). TDP-43 variants with increased aggregation propensities did not exhibit higher cytotoxicity (Figure 4A), in accord with previous results.^19^ Consistently, double mutants located in the droplet region of the landscape (*p*_DP_ > 0.85; MBM < 2.0; Methods) have increased cytotoxicity as compared to variants located in the amyloid region (*p*_DP_ < 0.75; MBM > 2.25) (Figure 4B). Then, we compared the cytotoxicity of the amyloid, hot-spot, and droplet regions using the classification based on the droplet landscape (see Methods), obtaining results that suggest that double mutations affecting droplet formation exhibit significantly higher cytotoxicity than those promoting amyloid formation or serve as aggregation hot-spots (Figure 4C). Our analysis also indicated that mutations increasing glycine (G) or proline (P) content also increase cytotoxicity, while those depleting these residues decrease cytotoxicity (Figure S1). This is in accord with previous results that G and P facilitate the self-organization of elastomeric sequences.^33^

These results are consistent with the conclusion that TDP-43 aggregation-promoting mutants may not provide major contributions to cytotoxicity. Instead, mutations affecting the droplet state and perturbing disordered interactions increase cytotoxicity.

### Extension of the FuzDrop Method to Predict the Cytotoxicity of Aging Condensates

The analysis reported above indicates that the molecular determinants of the condensation pathway offer insight into the cytotoxicity of the mutants. Thus, we probed whether we can quantitatively estimate the change in experimental cytotoxicity^19^ based on these quantities: the mutation-induced changes in droplet-forming (Δ*p*_DP_) and amyloid-forming (Δ*p*_AP_) probabilities as well as the change in MBM (ΔMBM). Droplet-promoting propensities (*p*_DP_) were computed by the FuzDrop program,^6^ and amyloid-promoting propensities (*p*_AP_) were obtained from the solubility scores by the CamSol program,^34^ and MBM values were derived from the FuzPred program.^22^ We determined the differences in these quantities for the mutant and the wild-type sequences (Methods).

**Figure 5:**
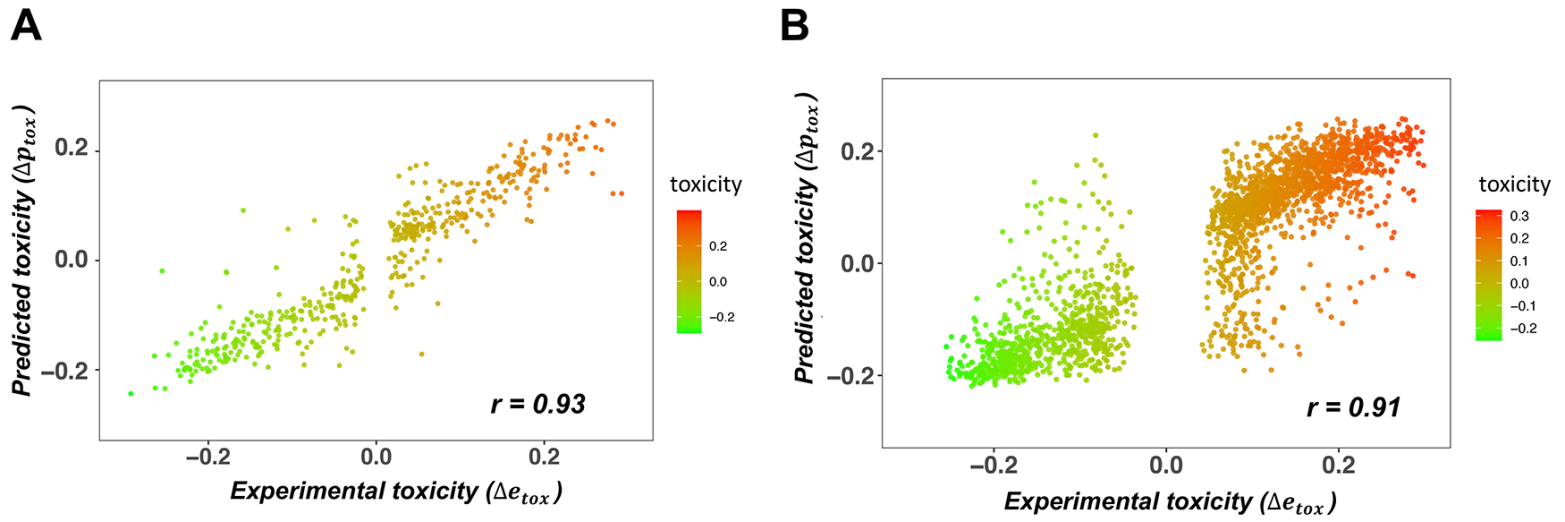
Correlation between experimental (Δ*e*_tox_) and predicted (Δ*p*_tox_) changes in cytotoxicity upon TDP-43 single (A) and double (B) missense mutants. Predictions were performed with the extended FuzDrop method based on three parameters: the change in droplet-promoting probability (Δ*p*_DP_), amyloid-promoting probability (Δ*p*_AP_), and change in multiplicity of binding modes (ΔMBM). (A) Prediction of the cytotoxicity of single mutants. Application of random forest models (Methods) on 498 single variants. (B) Prediction of the cytotoxicity of double mutants. Application of random forest models (Methods) on 23,802 double variants, where the parameters were averaged for the region of residues 312–341. Only 10% of the data is shown for clarity, and the *R* value is computed for the whole data set (Table 1). Pearson’s correlation coefficients were calculated in R. The cytotoxicity scale ranges from green (not toxic) to red (toxic) and is shown on the right panels. Cytotoxicity values were taken from ref (19).

We used optimized random forest approaches with the out-of-bag (OOB) validation technique (Methods) on Δ*p*_DP_, Δ*p*_AP_, and ΔMBM parameters used for TDP-43 single and double mutants with Δ*e*_tox_ ≥ 3σ ,^19^ respectively. We obtained Pearson’s correlation coefficient between the experimental (Δ*e*_tox_) and predicted cytotoxicity (Δ*p*_tox_) values of *r* = 0.975 for 498 single mutants and *r* = 0.983 for 23,802 double mutants (Table S3). Then, we developed a combined model for both single and double TDP-43 mutants (Methods). The combined model also gave a good performance, with *r* > 0.90 between the experimental and predicted cytotoxicity values of the different data sets (Table 1 and Figure 5). In particular, it also exhibited a comparable performance on the data set of droplet region mutations (Table 1), which provide a major contribution to cytotoxicity. Then, we applied the model to ALS-associated mutants^35^ (Table S4). Using 2430 double mutants, where at least one of the mutations was associated with ALS, we obtained Pearson’s correlation coefficient of *r* = 0.89 (Table 1). Although cytotoxicity of TDP-43 was assessed in a model organism,^19^ this analysis suggests the presence of general molecular mechanisms.

**Table 1:** Correlation between Experimental (Δ*e*_tox_) and Predicted (Δ*p*_tox_) Changes in Cytotoxicity^a^

mutations	data set	*N*	*R*
double	all	23,802	0.911
	droplet region	7,296	0.903
	ALS-associated	2,430	0.885
single	all	498	0.933
	droplet region	271	0.880

a Random forest models were developed on a combined set of single and double mutants, respectively. *N* is the size of the data set. Pearson’s correlation coefficients were computed by the R program.

Our analysis indicates that changes in cytotoxicity during droplet maturation can be predicted from the protein sequence based on the change in droplet-forming probability (Δ*p*_DP_), amyloid-forming probability (Δ*p*_AP_), and change in multiplicity of binding modes (ΔMBM).

## Discussion and Conclusions

The possibility for proteins of populating different states creates a challenge for the protein homeostasis system, since dysregulated transitions into nonfunctional assemblies can generate pathological processes.^12,16^ In particular, aging condensates often appear to cause cytotoxicity and to be associated with neurological disorders.^25,36,37^

In this study, we have investigated the amino acid code of the cytotoxicity of aging protein condensates. Our approach is based on the analysis of the binding modes in the droplet and amyloid states.^21,38^ Since changes in the multiplicity of binding modes due to sequence modifications (e.g., post-translational modifications) or cellular properties (e.g., localization) may enable proteins to switch between the different states,^5^ we reasoned that changes in binding modes may also affect interactions with cellular partners contributing to promiscuity .

Our analysis indicates that mutations promoting well-defined, ordered interactions and aggregation decrease cytotoxicity, in agreement with previous observations.^19^ In contrast, mutations that perturb disordered interactions and TDP-43 droplets tend to increase cytotoxicity. These observations are in accord with previous results that structurally labile regions (LARKs) are associated with TDP-43-linked pathologies.^31,32^ Earlier results also suggested that protein hydrogels may contain amyloid-like structures.^39,40^

In conclusion, our analysis suggests that the amino acid code for the cytotoxicity of aging droplets may be similar to that for the condensation pathway from the droplet to the amyloid states and that amyloid aggregation within condensates may have a partially protective role against cytotoxicity. It will be interesting to investigate whether these conclusions will extend beyond the case of TDP-43 investigated here.

## Methods

### Probability of Disordered Interactions

The probability of disordered interactions, *p*_DD_, is estimated for each amino *A_i_* as^20^
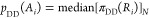
1where π_DD_(*R_i_*) is the probability of disordered binding mode of *R_i_*, a region of 5–9 residues around *A_i_*, and *N* is the number of possible regions *R_i_*. We refer to π_DD_(*R_i_*) as the “binding mode probability” of region *R_i_* because a value of 0 indicates a binding from fully disordered to fully ordered states and a value of 1 indicates a binding from fully disordered to fully disordered states. *p*_DD_ is computed using the FuzPred program.^20^

### Residue-Specific Multiplicity of Binding Modes (MBM)

The MBM was derived from the Shannon entropy of binding modes (*S*_bind_),^22^ which quantifies the variability of binding modes at the amino acid level. To define *S*_bind_, we start by defining the frequency *f* of different possible binding modes for an amino acid *A_i_*:
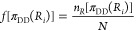
2To calculate *f*, the binding modes are divided into discrete bins (usually 10), and *n_R_* is the number of binding regions within a given bin. The Shannon entropy of binding modes (*S*_bind_) is then defined as the entropy of the frequencies of *f*:^22^
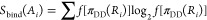
3where the summation is over the [π_DD_(*R_i_*)] bins.

### Residue-Specific Droplet-Promoting Probability

The droplet-promoting propensity profile *p*_DP_ quantifies the probability of spontaneous phase separation^6^
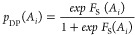
4where *F*_S_ (*A_i_*) is a scoring function for residue *A_i_*
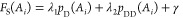
5Here, *p*_D_(*A_i_*) is the probability of disorder in the free state, and *p*_DD_(*A_i_*) is the probability of disordered binding. *p*_D_(*A_i_*) approximates the conformational entropy in the unbound form, while *p*_DD_(*A_i_*) estimates the conformational entropy of binding. λ_1_ and λ_2_ are the linear coefficients of the predictor variables and γ is a scalar constant (intercept), which were determined using the binary logistic model.^6^*p*_D_ was derived from the disorder score as computed using the ESpritz NMR algorithm.^41^ The *p*_DD_ values were predicted by the FuzPred method.^20^*p*_DP_ = 0.60 is the threshold used to predict whether a residue is readily involved in spontaneous phase separation.^6^

### Residue-Specific Amyloid-Promoting Probability

The amyloid-promoting propensity profile *p*_AP_ of a protein expresses its sequence-dependent probability to aggregate. Amyloid-promoting propensity profiles were obtained by the solubility profiles obtained by the CamSol program (*p*_CS_).^34^*p*_AP_ = – *p*_CS_ and *p*_AP_ = 0.90 is the threshold above which a protein is predicted to readily aggregate.^42^

### Analysis of TDP-43 Deep-Sequencing Data

We analyzed 498 single and 23,802 double TDP-43 missense mutants with Δ*e*_tox_ ≥ 3σ.^19^ Mutations were assigned to amyloid core (321–330 residues), droplet (262–311; 342–414), and “aggregation hot-spot” regions (312–320; 331–341) based on experimental data σ.^19^ For classification of double mutations, we used only both mutations in the same region. Droplet-promoting probabilities (*p*_DP_) were computed by the FuzDrop program,^6^ and amyloid-promoting propensities (*p*_AP_) were obtained from the solubility scores by the CamSol program.^34^ In the case of single mutants, we determined the differences in these quantities computed for the mutant and the wild-type (UniProt Q13148) residue. In the case of double mutants, we averaged the *p*_DP_, *p*_AP_, and MBM values for the 312–341 residue region and computed the difference between the average values of the mutant and wild-type sequence.

### Extending the FuzDrop Method to Predicting Cytotoxicity of Protein Droplets upon Mutations

We defined Δp_tox_ using a machine learning method with three input parameters : the difference between the mutant and wild-type protein in residue-specific droplet-promoting probability (Δ*p*_DP_) as computed by the original FuzDrop method,^6^ the change in residue-specific amyloid-promoting probability (Δ*p*_AP_) obtained as the negative of the solubility score of the CamSol program,^34^ and the change in multiplicity of binding modes (ΔMBM) obtained as the differences between the *S*_bind_ by the FuzPred method.^22^

The models were built using the random forest method with the OOB validation technique where two-thirds of the original data set is used for training and validation is performed on the remaining part. Random forest models with the highest Pearson’s correlation coefficients were inferred using grid optimization on the parameters of the number of individual decision trees (*ntree*) and the number of variables used at each split (*mtry*) with the randomForest package using the R program. Models were developed on single and double mutation data sets, respectively (Table S3), as well as using a combined data set of 498 single mutants and 23802 double mutants. In-house R scripts used to generate data and figures as well as the serialized random forest models can be downloaded from the GitHub repository (https://github.com/ahorvath/Biochemistry_2022.git).

The models were tested on all mutants with Δ*e*_tox_ ≥ 3σ, as well as on mutations of the droplet region (Table 1 and Table S3). In addition, the models were tested on mutations, where at least one of the mutations was ALS-associated^35^ (Table S4).

## Supporting Information Available

Characterization of the single and double TDP-43 mutants and ALS-associated mutants and the performance of the model (PDF)
Molecular determinants of the condensation pathway (XLSX)
Molecular determinants of the condensation pathway (XLSX)
Single and double mutation data sets (XLSX)
ALS-associated mutants (XLSX)
