# Supplementary material for: Sequence-based Prediction of the Cellular Toxicity Associated with Amyloid Aggregation within Protein Condensates
Source: Biochemistry. 2022 Nov 7;61(22):2461–9. doi: 10.1021/acs.biochem.2c00499 (PMC9670855; doi:10.1021/acs.biochem.2c00499)
Supplement: Supplementary file 1 — bi2c00499_si_001.pdf [file bi2c00499_si_001.pdf]

Supplementary Material

Sequence-based prediction of the cellular toxicity associated with amyloid aggregation within protein condensates

Attila Horvath[1], Michele Vendruscolo[2] and Monika Fuxreiter[3,4*]

[1]John Curtin School of Medical Research, The Australian National University, Acton, ACT 2601 Australia
[2]Centre for Misfolding Diseases, Yusuf Hamied Department of Chemistry, University of Cambridge, Cambridge CB2 1EW, UK
[3]Department of Biomedical Sciences, University of Padova, PD 35131, Italy
[4]Department of Physics and Astronomy, University of Padova, PD 35131, Italy

Supplementary Tables

Table S1. Comparison of droplet-forming probabilities, amyloid-forming probabilities and multiplicity of binding modes in single mutants of droplet, amyloid and aggregation hot-spot regions. Classification was based on the position of the mutation. Differences were obtained between the mutant and the wild-type parameters.
toxic: $\Delta e_{tox} > 0$
non-toxic: $\Delta e_{tox} \leq 0$
N: *number of mutants*
p_{AP} *: amyloid-promoting probability*
p_{DP} *: droplet-promoting probability*
MBM*: multiplicity of binding modes*
Δp_{AP} *: change in amyloid-promoting probability*
Δp_{DP} *: change in droplet-promoting probability*
ΔMBM*: change in multiplicity of binding modes*

Table S2. Comparison of droplet-forming probabilities, amyloid-forming probabilities and multiplicity of binding modes in double mutants of droplet, amyloid and aggregation hot-spot regions. Classification was based on the position of both mutations. Differences were obtained between the mutant and the wild-type parameters. The median of the distribution is shown.
toxic: $\Delta e_{tox} > 0$
non-toxic: $\Delta e_{tox} \leq 0$
N: *number of mutants*
p_{AP} *: amyloid-promoting probability*
p_{DP} *: droplet-promoting probability*
MBM*: multiplicity of binding modes*
Δp_{AP} *: change in amyloid-promoting probability*
Δp_{DP} *: change in droplet-promoting probability*
ΔMBM*: change in multiplicity of binding modes*

Table S3. Correlation between experimental (Δe_{tox}**) and predicted (**Δp_{tox}**) change in cytotoxicity upon TDP-43 single and double missense mutants.** Random Forest models were developed on single and double mutants, respectively. Pearson's correlation coefficients were computed by the R program. Models developed using mutations only in droplet region are marked as 'Drop'. The parameters of number of individual decision trees (*ntree*) and number of variables used at each split (*mtry*) in R with the randomForest package are displayed.

Table S4. Droplet-landscape characterisation of ALS-associated TDP-43 single (Sheet 1**) and double (**Sheet 2**) mutants.** In double mutants at least one of the mutations was ALS-associated. Differences were obtained between the mutant and the wild-type parameters. In case of double mutants, the values were averaged for the 312-341 region.

MUT: *type and position of the mutation(s)*
TOX: *measured cytotoxicity (*$\Delta \boldsymbol{e}_{tox}$*)*
SD: *experimental std of cytotoxicity*
$\Delta \boldsymbol{p}_{AP}$: *change in amyloid-promoting probability*
$\Delta \boldsymbol{p}_{DP}$: *change in droplet-promoting probability*
ΔMBM: *change in multiplicity of binding modes*
$\boldsymbol{p}_{AP}$: *amyloid-promoting probability*
$\boldsymbol{p}_{DP}$: *droplet-promoting probability*
MBM: *multiplicity of binding modes*

Supplementary Figures

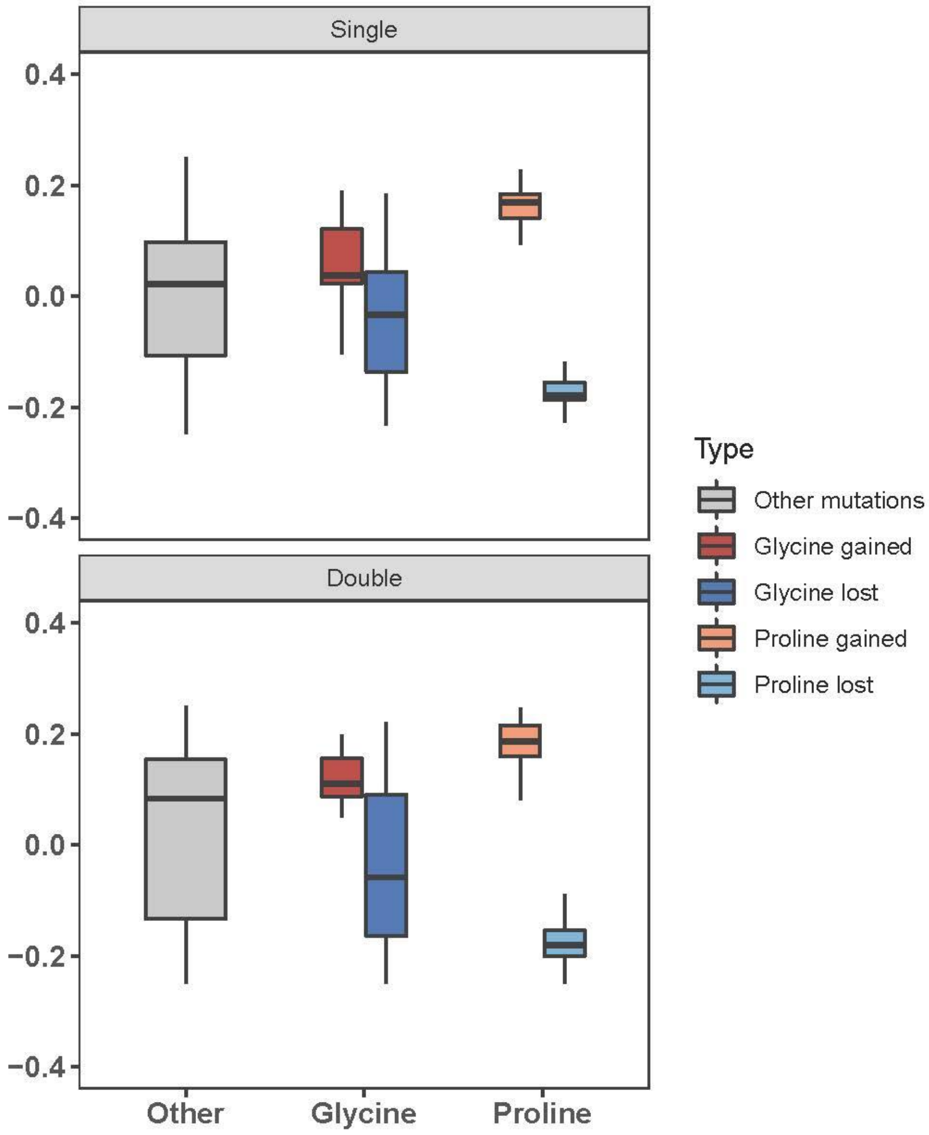

Figure S1. Enrichment in glycine and proline increase cytotoxicity. (A) Changes in glycine and proline compositions in single TDP-43 mutations. Mutations increasing G and P (red) increase cytotoxicity (Δe_{tox}~0.05), whereas mutations leading to depletion of these two residues (blue) significantly decreased cytotoxicity (Δe_{tox}~-0.2). Other mutations (gray) have a smaller impact on cytotoxicity. **(B) Changes in glycine and proline compositions in double TDP-43 mutations.** Mutations increasing G and P (orange) increase significantly increase cytotoxicity, whereas mutations leading to depletion of these two residues (cyan) decrease cytotoxicity. Other mutations (gray) have decreased impact on cytotoxicity. Statistical analysis was performed using a Mann-Whitney test of the R program, all deviations between increased and decreased G/P content are significant.
